# Vaccination Against Serogroup B Meningococcal Disease: Current Status and Future Perspectives—A Consensus Document of the World Association for Infectious Diseases and Immunological Disorders (WAidid)

**DOI:** 10.3390/vaccines14060502

**Published:** 2026-06-03

**Authors:** Susanna Esposito, Nigel Curtis, Ulrich Heininger, Markus Knuf, Shamez Ladhani, Helen Marshall, Federico Martinon-Torres, Marco Safadi, Vana Spoulou, Mohamed K. Taha, Nicola Principi

**Affiliations:** 1 Pediatric Clinic, University Hospital of Parma, 43126 Parma, Italy; 2 Department of Paediatrics, The University of Melbourne, Parkville, VIC 3052, Australia; nigel.curtis@rch.org.au; 3 Murdoch Children’s Research Institute, Royal Children’s Hospital, Parkville, VIC 3052, Australia; 4 Division of Pediatric Infectious Diseases and Vaccinology, University Children’s Hospital Basel (UKBB), Spitalstrasse 33, 4056 Basel, Switzerland; ulrich.heininger@ukbb.ch; 5 Department of Pediatrics, Worms Clinic, 67550 Worms, Germany; markus.knuf@klinikum-worms.de; 6 Pediatric Infectious Diseases, University Medicine Mainz, 55101 Mainz, Germany; 7 Immunisation and Countermeasures Division, UK Health Security Agency & St George’s, University of London, London SW17 0RE, UK; shamez.ladhani@ukhsa.gov.uk; 8 Women’s and Children’s Health Network, North Adelaide, SA 5006, Australia; helen.marshall@adelaide.edu.au; 9 The Robinson Research Institute, Adelaide Medical School, Adelaide University, North Adelaide, SA 5005, Australia; 10 Translational Pediatrics and Infectious Diseases, Hospital Clínico Universitario de Santiago & Universidad de Santiago de Compostela, 15706 Santiago de Compostela, Spain; federico.martinon.torres@sergas.es; 11 Department of Pediatrics, Santa Casa de São Paulo School of Medical Sciences, Rua Dr. Cesário Motta Júnior 112, São Paulo 01221-020, SP, Brazil; masafadi@uol.com.br; 12 First Department of Pediatrics, National and Kapodistrian “Agia Sophia” Children’s Hospital, Thivon & Papadiamantopoulou, University of Athens, 11527 Athens, Greece; vspoulou@med.uoa.gr; 13 National Reference Center for Meningococci, Institut Pasteur, 75724 Paris, France; muhamed-kheir.taha@pasteur.fr; 14 Department of Pathophysiology and Transplantation, Università degli Studi di Milano, 20122 Milan, Italy; nicola.principi@unimi.it

**Keywords:** meningococcal disease, *Neisseria meningitidis*, serogroup B, 4CMenB vaccine, vaccine effectiveness, immunization strategies

## Abstract

Background: Invasive meningococcal disease (IMD) remains a rare but severe condition associated with high mortality and a significant risk of long-term sequelae. Despite global vaccination efforts, the epidemiology of *Neisseria meningitidis* continues to evolve, with serogroup B (MenB) representing the predominant cause of IMD in many high-income countries. Methods: This consensus document reviews current evidence on MenB epidemiology and the role of the multicomponent meningococcal serogroup B vaccine (4CMenB), with a focus on immunogenicity, strain coverage, real-world effectiveness, and remaining challenges. Results: Protein-based MenB vaccines have overcome the limitations of polysaccharide approaches, demonstrating robust immunogenicity across age groups. Real-world data confirm substantial vaccine effectiveness, particularly in infant immunization programs and outbreak settings, with significant reductions in disease incidence. For example, in England in the 3 years after vaccine introduction, MenB IMD incidence declined by 75% in immunized infants compared to unvaccinated controls. Adjusted vaccine efficacy was 52.7% after the two-dose primary series and 59.1% following the booster dose, highlighting the contribution of the booster. However, protection is influenced by antigenic variability among circulating strains, resulting in incomplete and geographically variable coverage. In addition, antibody waning over time and the limited impact on nasopharyngeal carriage reduce the potential for long-term and indirect protection. These factors highlight the need to optimize vaccination strategies, including the timing of booster doses, particularly in adolescents, and the role of vaccination in different epidemiological contexts. In this regard, it is not precisely defined whether infants who were immunized in the first year of life need a booster dose in the preschool period, especially in countries with a high incidence of MenB disease. Moreover, it is not established whether and when adolescents who were vaccinated both in infancy and during the preschool period need a booster dose. Economic considerations and variability in national immunization policies further contribute to heterogeneity in vaccine implementation. Emerging evidence suggests possible cross-protection against other meningococcal serogroups and *Neisseria gonorrhoeae*, although findings remain inconsistent across different risk groups and do not allow us to recommend 4CMenB vaccine beyond MenB IBD prevention. Conclusions: 4CMenB is an effective tool for preventing MenB IMD, although further studies are needed. Future strategies should prioritize age-targeted boosting and enhanced genomic surveillance to maximize impact.

## 1. Introduction

*Neisseria meningitidis* (Men) is an encapsulated, aerobic, Gram-negative diplococcus belonging to the *Neisseriaceae* family and remains a leading cause of bacterial meningitis and sepsis worldwide. It is classified into 12 serogroups based on the antigenic structure of its polysaccharide capsule, of which six—A, B, C, X, Y, and W—account for the vast majority of invasive meningococcal disease (IMD). While serogroup X circulates predominantly in sub-Saharan Africa, the other major serogroups are distributed globally, with marked geographic variability in their prevalence [1]. In addition to capsular classification, Men is further characterized by multilocus sequence typing into clonal complexes (CCs), which group strains according to genetic relatedness and virulence potential. A limited number of hypervirulent lineages—including CC11, CC32, CC41/44, CC269, and CC23—capable of bypassing human immune defenses and cause rapid, life-threatening invasive disease are responsible for most IMD cases and outbreaks, with specific associations between CCs and serogroups (e.g., CC11 with serogroups C and W, CC32/41/44/269 with serogroup B, and CC23 with serogroup Y) [2].

Although IMD is relatively rare in high-income countries, where annual incidence is typically <1 case per 100,000 population, its clinical and public health impact remains substantial. In 2023, 1895 confirmed cases were reported in the EU/EEA (0.4 per 100,000 population) [3]. In contrast, the burden is considerably higher in low-resource settings, particularly in the African meningitis belt, where approximately 30,000 cases occur annually, and incidence during epidemics may reach up to 1000 cases per 100,000 population [4]. Clinically, IMD classically presents as meningitis, septicemia, or both; however, up to 20% of cases may manifest with atypical, extra-meningeal presentations such as pneumonia, abdominal syndromes, or arthritis [5,6]. Despite advances in antimicrobial therapy, case-fatality rates remain high. It varies from 20% to 40% in cases of sepsis and is about 5% when meningitis occurs. When both conditions occur simultaneously, the mortality rate is typically between 10% and 12%. Moreover, 10–20% of survivors of sepsis experience severe long-term sequelae, including neurological impairment, hearing loss, and limb amputations [7,8].

Given this significant morbidity and mortality, prevention through vaccination has been a major public health priority. Early polysaccharide vaccines targeting serogroups A, C, W, and Y were limited by poor immunogenicity in young children and lack of immunological memory [9]. The subsequent development of conjugate vaccines, in which capsular polysaccharides are linked to carrier proteins, represented a major breakthrough, enabling robust immune responses across all age groups, reduction in nasopharyngeal carriage, and induction of herd protection [10]. These vaccines have been successfully implemented in national immunization programs and have dramatically reduced disease incidence caused by targeted serogroups [11,12]. The emergence of non-vaccine serogroups further prompted the adoption of quadrivalent MenACWY conjugate vaccines in many countries.

In contrast, the development of vaccines against serogroup B (MenB) posed unique challenges. The MenB capsular polysaccharide is poorly immunogenic and structurally similar to human neural cell adhesion molecules, raising concerns about autoimmunity [13]. This limitation led to the development of protein-based MenB vaccines, which include surface-exposed antigens, alone or combined with outer membrane vesicles (OMVs), to elicit protective immune responses [14]. More recently, pentavalent vaccines covering five major serogroups have been licensed, further expanding preventive strategies [15].

Despite these advances, important uncertainties remain regarding the optimal use of MenB vaccines. The epidemiology of MenB disease is highly dynamic, varying across regions and over time, with sporadic outbreaks interspersed with periods of low incidence. Consequently, it remains unclear whether MenB vaccination should be primarily targeted for outbreak control or integrated into routine immunization schedules. In addition, key questions persist regarding real-world vaccine effectiveness and safety, duration of protection and the need for booster doses, as well as the potential impact on nasopharyngeal carriage and herd immunity.

In this context, the aim of this consensus document is to critically evaluate the current evidence on the epidemiology of MenB disease and the performance of available MenB vaccines, with a particular focus on immunogenicity, effectiveness, strain coverage, and their implications for vaccination strategies and public health policy.

## 2. Methods

A structured literature search was conducted in PubMed and Embase considering studies published from 1 January 2000 to 3 January 2026, supplemented by manual review of surveillance reports from ECDC, UKHSA, and CDC. The following search terms were used: 4CMenB OR MenB-4C OR Bexsero OR meningococcal serogroup B OR meningococcal B vaccine OR meningococcus group B OR meningococcal group B OR B invasive meningococcal OR B-IMD OR serogroup B meningococcal immunization OR serogroup B vaccine OR meningococcal B recombinant vaccine AND reactogenicity OR surveillance OR adverse events OR safety OR *Neisseria meningitidis* OR adverse reactions OR monitoring OR pharmacovigilance OR Kawasaki OR nephrotic OR seizure OR tolerability OR tolerated. The search was carried out using the following filters: Books and Documents, Clinical Study, Clinical Trial, Phase II, Phase III, Comment, Congress, Evaluation Study, Meta-Analysis, Multicenter Study, Observational Study, Practice Guideline, Randomized Controlled Trial, Review, Systematic Review, and in the English language. Studies were included if they addressed MenB epidemiology, 4CMenB immunogenicity, effectiveness, safety, and strain coverage. The selection and weighting of evidence, including conflicting findings, reflect expert judgment. Consensus among the authors was achieved through iterative review and discussion.

## 3. Invasive Meningococcal Epidemiology

The epidemiology of IMD is highly dynamic and varies substantially by age, geography, and time. Incidence consistently peaks in infants (<1 year of age), with secondary increases observed among adolescents and young adults, and, more recently, among older adults (≥65 years). In Europe, for example, in 2023, the highest notification rate was reported in infants (6.5 cases per 100,000 population), followed by children aged 1–4 years (1.2 per 100,000). Incidence was lower but still notable among adolescents and young adults aged 15–24 years (0.8 per 100,000), older adults ≥65 years (0.4 per 100,000), and children aged 5–14 years (0.3 per 100,000) [3].

In addition to age, specific populations are at markedly increased risk of IMD. These include individuals with complement deficiencies or receiving complement inhibitors (e.g., for atypical hemolytic uremic syndrome or paroxysmal nocturnal hemoglobinuria, as well as other increasing indications for anti-complement drugs), those who underwent hematopoietic stem cell transplantation, persons with functional or anatomic asplenia, microbiologists with occupational exposure, individuals during outbreak settings, and those living in high-density environments, such as military recruits and university students.

Temporal trends in IMD are shaped by multiple interacting factors, including changes in surveillance and case definitions, the emergence and spread of hypervirulent clonal complexes, population behavioral patterns, environmental conditions, and the implementation of vaccination programs. These determinants contribute to pronounced regional variability in both overall incidence and the distribution of circulating serogroups. A notable example is the near elimination of serogroup C disease in many high-income countries and serogroup A disease in the African meningitis belt following the introduction of targeted vaccination programs [16].

Recent environmental and societal changes have further influenced IMD epidemiology. The marked global decline observed during the COVID-19 pandemic coincided with widespread non-pharmaceutical interventions, such as lockdowns and mask use, which reduced transmission of respiratory pathogens [17]. Following the relaxation of these measures, a rebound in IMD incidence has been observed in several regions. This resurgence has been partly attributed to reduced population-level exposure to pathogens—so-called “immunity debt”—resulting in a larger pool of susceptible individuals. Changes in vaccine uptake and disruptions to immunization programs during the pandemic may have also contributed to shifts in serogroup distribution [18]. Interpretation of epidemiological trends, however, remains challenging due to heterogeneity in case definitions and surveillance systems across countries [19].

### Invasive Meningococcal B Disease Epidemiology

Prior to the COVID-19 pandemic, the global incidence of MenB IMD showed an overall declining trend, although with marked regional heterogeneity [20]. MenB disease was largely absent across most of Africa (with the exception of South Africa) and occurred only sporadically in parts of Asia, including China and India. In contrast, low-level endemic transmission persisted in Europe, North America, South America, and Australia, typically with incidence rates below one case per 100,000 population.

In Europe, MenB incidence declined from 0.69 per 100,000 in 2008 to 0.30 per 100,000 in 2017, with a corresponding decrease in its contribution to overall IMD cases from 71.5% to 48.0% [21]. Nevertheless, MenB remained the predominant cause of IMD in many high-income settings, including Europe and the United States [22,23]. Only a few countries, notably New Zealand and Ireland, reported higher endemic incidence (>2 cases per 100,000 population). Globally, most MenB disease has been associated with a limited number of hyperinvasive clonal complexes, particularly CC32, CC41/44, and CC269, although regional variations have been described (e.g., CC865 in Argentina and contributions from CC35 in Europe and the United States).

From 2015 onwards, IMD incidence continued to decline in several countries, particularly where MenB vaccination programs were implemented. In England, the introduction of infant vaccination was associated with a rapid reduction of approximately 50% in MenB cases within the first year, irrespective of predicted strain coverage [23].

The COVID-19 pandemic led to a further transient decline in MenB incidence, likely driven by reduced transmission, although Men carriage prevalence did not decrease. In England, overall IMD incidence fell to 0.16 per 100,000 population in 2020–2021, with MenB accounting for 75% of cases. Following the relaxation of public health measures, incidence increased again to 0.27 per 100,000, with MenB representing 89.2% of cases. The resurgence initially affected adolescents and young adults—exceeding pre-pandemic levels—and subsequently extended to other age groups. Reported MenB cases in England increased from 61 in 2020/21 to 179 in 2021/22, 356 in 2022/23, and 301 in 2023/24.

Similar post-pandemic rebounds have been observed in multiple regions, including Europe, South America, and Australia, where MenB has been the principal driver of re-emergence [24]. In contrast, in some countries, the increase has been driven by other serogroups—for example, serogroups C, Y, and nongroupable strains in the United States, and serogroups Y and W in France and Chile [18,25]. Moreover, new clonal complexes seem to increase in the post-pandemic period, such as CC9316.

Differences in post-pandemic epidemiological patterns may, in part, reflect pre-existing vaccination policies and coverage. Countries with broader MenB vaccination strategies, including in adolescents or multiple age cohorts, appear to have experienced different age-specific trends compared with those where vaccination was primarily limited to infants, leaving older age groups more susceptible to renewed transmission [25]. These patterns indicate that MenB epidemiology is increasingly driven by dynamic transmission in adolescents and young adults, rather than stable endemic circulation.

To provide a structured overview of the key determinants and trends of IMD and MenB epidemiology, the main epidemiological features across different domains are summarized in Table 1.

## 4. The 4CMenB Vaccine

The multicomponent meningococcal serogroup B vaccine (4CMenB) is composed of three recombinant protein antigens—factor H binding protein (fHbp) variant 1.1, Neisseria adhesin A (NadA), and Neisserial heparin-binding antigen (NHBA)—combined with outer membrane vesicles (OMVs) derived from the New Zealand epidemic strain NZ98/254, which expresses Porin A (PorA) as the immunodominant antigen [26]. This antigenic combination is designed to broaden strain coverage by targeting multiple surface-exposed proteins.

4CMenB received regulatory approval in the EU/EEA in 2013 and has since been licensed in numerous countries worldwide. It is currently authorized in more than 50 countries, generally for use in infants from ≥2 months of age through adulthood [27]. In the United States, approval is limited to individuals aged 10–25 years [28].

Immunization policies for 4CMenB vary considerably across countries, reflecting differences in local epidemiology, public health priorities, and national advisory recommendations. Strategies range from universal infant immunization programs to targeted approaches focusing on adolescents or specific high-risk groups. Economic considerations, including cost-effectiveness assessments, have also played a key role in shaping implementation strategies and determining target populations.

In countries adopting infant vaccination, the most commonly used schedule is a 2 + 1 regimen (two primary doses followed by a booster). An exception is Italy, where a 3 + 1 schedule is recommended and included in the National Immunization Program. In older children and adolescents, vaccination is typically administered as a two-dose series. For individuals at increased risk, current practice generally involves a two-dose primary series followed by a booster after one year, with subsequent periodic boosters as long as the elevated risk persists.

In the United States, 4CMenB vaccination is recommended for adolescents aged 16–23 years (preferably 16–18 years) based on shared clinical decision-making, as well as for individuals aged ≥10 years who are at increased risk of meningococcal disease (Table 2).

### 4.1. Evaluation of Potential Coverage Offered by 4CMenB Vaccine

Vaccine efficacy is traditionally established by demonstrating a reduction in disease incidence in vaccinated compared with unvaccinated populations under controlled conditions, such as randomized clinical trials. However, this approach is not feasible for MenB vaccines due to the low incidence of IMD. Consequently, licensure of MenB vaccines has relied on immunogenicity and safety data. Specifically, regulatory approval was based on evidence that vaccination induces human complement-mediated serum bactericidal antibody (hSBA) responses at or above a predefined correlate of protection against a panel of representative, epidemiologically relevant MenB strains [29].

An hSBA titer ≥1:4 has been widely accepted as a correlate of protection, based on historical evidence demonstrating its association with protection against serogroup C IMD and its correlation with the effectiveness of outer membrane vesicle (OMV)-based vaccines [30,31].

For 4CMenB, early immunogenicity studies used panels of 7–15 reference strains selected to reflect genetic diversity, geographic and temporal distribution, and major hyperinvasive clonal complexes. Subsequently, the single-antigen indicator strains used to attribute bactericidal activity to individual vaccine components (44/76-SL for fHbp, 5/99 for NadA, NZ98/254 for PorA P1.4, and M10713 for NHBA) were selected or engineered to express only one vaccine-targeted antigen, which optimizes measurable hSBA responses relative to heterogeneous circulating strains that may co-express variant antigens at lower levels [32].

Despite robust immunogenicity, the breadth of protection conferred by 4CMenB is inherently constrained by the antigenic diversity and variable surface expression of MenB proteins. High hSBA responses against selected reference strains do not necessarily translate into universal protection, as circulating isolates may lack vaccine antigens or express divergent variants [33].

To better estimate potential strain coverage, several phenotypic and genotypic predictive tools have been developed [34]. The Meningococcal Antigen Typing System (MATS) assesses antigen presence and relative expression of fHbp, NHBA, and NadA, as well as PorA subtype; a strain is considered covered if the antibody titer against at least one antigen exceeds a predefined bactericidal threshold or if antibodies against PorA P1.4 are present. MATS has been validated as a conservative surrogate of hSBA but requires cultured isolates and specialized laboratory capacity [34].

The genetic MATS (gMATS) approach predicts MATS results from sequence data by linking antigen genotypes to empirically derived coverage thresholds. Calibration using large, multinational isolate collections has demonstrated strong concordance with phenotypic MATS results [35]. Estimates of 4CMenB coverage based on MATS/gMATS vary widely by region (generally 60–90% overall; ~58–88% in Europe and ~85% in the United States) [36,37,38,39], as well as by age group (typically lower in infants and young children) [36] and clonal complex (higher for CC32 and CC41/44, lower for others such as CC213) [40,41,42,43].

Additional approaches, such as the Bexsero Antigen Sequence Type (BAST) system, integrate antigen sequence data with clonal lineage to model coverage [34]. However, their accuracy depends on the completeness of reference datasets and may be limited when novel or poorly characterized antigen variants are present.

For fHbp-based vaccines (MenB-FHbp), complementary methods have been developed to assess strain susceptibility. The MEASURE assay quantifies surface expression of fHbp using flow cytometry; isolates with mean fluorescence intensity above defined thresholds are generally susceptible to vaccine-induced bactericidal antibodies [44,45]. Although fHbp is present in nearly all circulating MenB strains and is expressed at sufficient levels in over 90% of invasive isolates [46], MEASURE evaluates expression but does not account for antigenic sequence diversity or predict the proportion of vaccinees achieving protective responses. Furthermore, it is not applicable to PCR-confirmed cases without cultured isolates [47].

In summary, regulatory approval of MenB vaccines has relied on validated immunologic surrogates (hSBA) and safety data, complemented by predictive laboratory tools (MATS, gMATS, BAST, and MEASURE) to estimate potential strain coverage. Importantly, these tools provide probabilistic rather than definitive estimates of protection and may underestimate real-world effectiveness. While these approaches provide valuable and complementary insights, each has inherent limitations related to antigenic diversity, variability in expression, assay standardization, and representativeness of reference datasets. Accordingly, the apparent concordance between immunogenicity data based on hSBA responses, predictive coverage estimates generated by MATS/gMATS, and real-world effectiveness findings should be interpreted with caution. Although these evidence streams are complementary, they are not fully independent, because MATS and gMATS were calibrated against bactericidal activity and reference strain frameworks that also underpin immunogenicity-based assessment. Therefore, the consistency between hSBA results, predicted strain coverage, and observed vaccine effectiveness partly reflects shared methodological assumptions rather than entirely independent validation. Definitive confirmation of breadth of protection would require hSBA testing against stratified panels of unselected, contemporary wild-type MenB isolates representative of circulating clonal complexes, antigenic variants, and geographic diversity. Ongoing molecular surveillance and post-licensure effectiveness studies therefore remain essential to confirm real-world vaccine impact and to inform immunization strategies.

### 4.2. Immunogenicity and Safety of the 4CMenB Vaccine

Multiple clinical studies have evaluated the immunogenicity of 4CMenB across different age groups, particularly in infancy. Early phase 2 and phase 3 trials assessed a 3 + 1 infant schedule (primary series initiated at 2 months of age followed by a booster at 12 months) and demonstrated robust bactericidal responses against reference strains selected to represent individual vaccine antigens [48,49,50]. In one pivotal study, 100% of participants achieved protective hSBA titers against fHbp- and NadA-specific strains, while 84% reached protective titers against the PorA-containing OMV strain. Although antibody levels declined after the primary series, they were restored and strengthened following the booster dose, with 95–100% of children achieving hSBA titers ≥ 5 for all tested antigens [51].

Subsequent studies explored reduced schedules to enhance programmatic feasibility. In a randomized phase 3b trial, infants receiving 2 + 1 schedules (either 3½–5–11 months or 6–8–11 months) achieved high levels of seroprotection one month after the primary series: 98–100% for fHbp, NadA, and PorA, and 48–77% for NHBA [52]. As observed with the 3 + 1 regimen, antibody titers declined between the primary series and the booster but were restored following booster administration, indicating the induction of immunologic memory [53].

Long-term follow-up studies have consistently shown antigen-specific waning of antibody responses after completion of the infant schedule. In children primed with a 3 + 1 regimen, 36 months after the booster, the proportion with hSBA titers ≥ 4 remained high for NadA (90–93%) and moderate for NHBA (54–68%), but was substantially lower for fHbp (≈12%) and PorA (9–10%). Pooled analyses including both 2 + 1 and 3 + 1 schedules confirm this pattern: although antibody levels suggesting protection are high shortly after the booster (fHbp 87–100%, NadA 95–100%, PorA 70–100%, and NHBA 49–97%), they decline over time, with marked variability across antigens. At 12 months post-booster, persistence is approximately 62% for fHbp, 97% for NadA, 17% for PorA, and 36% for NHBA, with further decline observed at 24–36 months [54,55,56,57].

These findings indicate that NadA-specific responses are the most durable, whereas fHbp and PorA responses wane more rapidly. This antigen-specific decline suggests that a proportion of children may have suboptimal protection during the preschool years. Accordingly, administration of a booster dose at 3–4 years of age has been shown to elicit strong anamnestic responses, restoring high hSBA titers across all antigens regardless of the primary schedule. Pooled data indicate post-booster seroprotection rates of approximately 97–100% for fHbp and NadA, 83–100% for PorA, and 75–100% for NHBA [52,53,55,56]. These results support the capacity of 4CMenB to induce durable immune memory and highlight the potential role of preschool boosters, particularly in individuals at increased risk.

Similar considerations apply to children who initiate vaccination after the first year of life. Studies have shown that two-dose schedules in toddlers (12–30 months) do not consistently provide long-term protection against all antigens, with substantial waning observed over time. However, a booster dose effectively restores protective antibody levels in the majority of recipients [58].

In older children and adolescents, two-dose schedules elicit strong initial immune responses [59]. In preschool and school-age children, 24–36 months after vaccination, protective hSBA titers (≥4) are maintained in 52–58% for fHbp, 79–85% for NadA, 29–50% for PorA, and 42–66% for NHBA [56]. In adolescents, two doses administered 1–6 months apart induce near-universal bactericidal responses, with more than 60% of individuals retaining protective titers at 18–24 months [60]. Although antibody levels decline over time, persistence of protection—particularly for NadA and NHBA—has been demonstrated up to 4–7.5 years after vaccination [61].

The potential need for booster doses in adolescence depends, in part, on prior vaccination history. In individuals primed during infancy, booster responses in adolescence vary according to the timing and number of earlier doses. Rollier et al. [62] showed that adolescents who had received both infant and preschool doses mounted stronger responses to a booster compared with vaccine-naïve individuals, whereas those primed only in infancy exhibited weaker responses, suggesting suboptimal long-term memory when early schedules are not reinforced. These findings support consideration of booster strategies in individuals primed in infancy without subsequent doses.

Regarding safety, both clinical trial and post-licensure data indicate that 4CMenB is associated with a relatively high frequency of reactogenicity, although adverse events are predominantly mild to moderate and self-limiting. Local reactions occur in approximately 25–45% of recipients and are often accompanied by systemic symptoms, including irritability (up to 75%), fatigue (51–59%), headache (42–49%), and persistent crying (50–65%) [63]. Fever is the most clinically relevant systemic reaction, particularly in infants, among whom up to 60% develop fever within 24–72 h after the first doses, especially when co-administered with routine vaccines [64].

To mitigate reactogenicity, health authorities such as the EMA recommend prophylactic administration of paracetamol and, when feasible, separation from other routine vaccinations [65]. Available evidence indicates that these strategies do not impair immunogenicity. Real-world data further support their effectiveness: in a national audit in England, fever > 38 °C occurred in 20% of preterm infants who did not receive prophylactic paracetamol, compared with only 7% among those who did [66].

Overall, 4CMenB demonstrates strong immunogenicity across age groups, the ability to induce immunologic memory, and an acceptable safety profile, although antigen-specific waning and reactogenicity remain important considerations for optimizing vaccination schedules. The key findings on immunogenicity, antibody persistence, and safety of the 4CMenB vaccine across different age groups and vaccination schedules are summarized in Table 3.

### 4.3. Real-World Effectiveness

Despite heterogeneity across studies evaluating real-world vaccine effectiveness (VE) and population impact (VI), with substantial variation in study design, target age groups, vaccination schedules, background incidence, and circulating strain distribution, post-licensure evidence accumulated over the past decade has consistently provided robust support for the effectiveness and public health impact of the 4CMenB vaccine. Overall, these findings are consistent with earlier predictions based on MATS and gMATS analyses, confirming that 4CMenB confers substantial protection against IMD for several years following completion of the vaccination schedule [67,68,69,70,71,72,73,74].

Most real-world evidence derives from infant immunization programs implemented in high-income countries. In England, where vaccine uptake exceeded 90% following introduction of a 2 + 1 infant schedule, both VE and VI were assessed during the first three years of program implementation [74]. VE was estimated by comparing disease incidence in vaccinated versus unvaccinated children, while VI was calculated by comparing observed cases after vaccine introduction with those expected based on historical surveillance data. Integration of MATS-derived coverage estimates further enabled identification of vaccine failures.

Between 2015 and 2018, MenB incidence in vaccine-eligible cohorts declined markedly, corresponding to a 75% reduction. Adjusted VE was 52.7% after the two-dose primary series and 59.1% (95% CI, −31.1 to 87.2) following the booster dose [75], highlighting both the contribution of the booster and the sustained protection afforded by the schedule. Overall, 169 cases were observed compared with the 446 expected, corresponding to 277 prevented cases, with only 21 previously vaccinated children among approximately two million vaccinated children. Longer-term analyses have confirmed the persistence of these effects beyond five years. Notably, implementation of infant vaccination was also associated with a reduction in the mean age of cases, underscoring the importance of early protection [76].

Evidence from Italy further supports the benefit of early vaccination. Although VE was similarly high (>90%) across regions using different schedules, VI varied depending on the timing of the first dose. Infants vaccinated starting at 2 months experienced a 68% reduction in cases, compared with 31% among those vaccinated from 7 months of age. Additionally, approximately 20% of IMD cases in unvaccinated children occurred in infants too young to be vaccinated, reinforcing the importance of early immunization [71].

Data from Spain also confirm substantial effectiveness. In a nationwide case–control study including children <5 years of age, complete vaccination conferred 71% effectiveness against MenB IMD and 76% against IMD overall, while partial vaccination (≥1 dose) was associated with 64% effectiveness against MenB disease. Importantly, none of the MenB cases caused by strains predicted to be covered by 4CMenB occurred in vaccinated individuals, supporting the biological plausibility of protection [73].

Although data in adolescents are more limited, available evidence suggests that vaccine effectiveness remains high in this age group. In South Australia, implementation of an adolescent immunization program was associated with a 71% reduction in MenB incidence, and a vaccine effectiveness of 83.5% in subsequent evaluations [77,78].

Waning of functional antibodies over time likely limits the duration of individual protection, particularly in the absence of booster doses. Consequently, the overall population impact of vaccination depends on multiple factors, including timing of immunization relative to age-specific risk, vaccine coverage, and the epidemiology of circulating strains.

Additional evidence of effectiveness comes from outbreak settings. In Canada, a mass vaccination campaign targeting individuals ≤20 years of age during a prolonged outbreak caused by a hypervirulent ST-269 clone achieved 83% coverage and resulted in a dramatic decline in incidence, from 11.4 to 0.4 cases per 100,000 population (*p* < 0.0001) [67]. Similarly, vaccination campaigns in US university settings successfully interrupted transmission, with no further cases reported following completion of two-dose schedules [78,79].

In contrast to its strong direct effects, the impact of 4CMenB on nasopharyngeal carriage—and thus on herd immunity—appears limited and inconsistent. Studies in adolescents have generally failed to demonstrate significant reductions in carriage prevalence or density following vaccination. For example, no difference in meningococcal carriage density was observed between vaccinated and unvaccinated adolescents in South Australia, and no correlation between hSBA responses and carriage was found in a randomized trial among university students [80]. Similarly, population-level analyses in Canada did not demonstrate indirect protection in unvaccinated age groups, suggesting that observed declines were not mediated by reduced transmission [67].

Taken together, these findings suggest that 4CMenB behaves as a primarily individual-protection vaccine, with limited population-level transmission effects.

Beyond protection against MenB disease, 4CMenB may confer cross-protection against other meningococcal serogroups and *Neisseria* species due to shared protein antigens [81]. Surveillance data from England have shown a 69% reduction in MenW disease among vaccine-eligible infants following 4CMenB implementation [74]. In vitro and immunologic studies further suggest potential activity against MenY and MenX strains [82,83].

Finally, 4CMenB has been investigated for its potential effect against *Neisseria gonorrhoeae*. Observational studies have reported moderate short-term protection (approximately 30%), with declining effectiveness over time [84,85]. These findings prompted the introduction of targeted vaccination programs in high-risk populations in some settings, such as the United Kingdom in 2025 [86]. However, more recent randomized trial data in those at very high risk of gonorrhea show no significant reduction in gonorrhea incidence (VE −0.5%; 95% CI, −26.2% to 19.9%) [87]. Accordingly, no recommendation can currently be made for the use of the 4CMenB vaccine beyond the prevention of IMD.

Overall, these findings suggest that 4CMenB behaves as a primarily individual-protection vaccine against MenB, with limited population-level transmission effects. Its impact on non-B and other *Neisseria* species remains variable and requires further investigation. The main findings from real-world studies evaluating the effectiveness and impact of 4CMenB across different settings and populations are summarized in Table 4.

## 5. When to Administer a Booster in Adolescence

The optimal timing of a 4CMenB booster dose in adolescence remains an area of ongoing investigation, but available immunologic and epidemiologic evidence provides a strong rationale for targeted strategies. Adolescence represents a secondary peak in the incidence of IMD, associated with increased social mixing and higher nasopharyngeal carriage rates [88]. At the same time, individuals primed in infancy often show waning bactericidal antibody levels by late childhood, particularly against certain vaccine antigens such as factor H binding protein (fHbp) and PorA [89]. This combination of declining immunity and increased exposure risk supports consideration of a booster dose during adolescence.

Immunogenicity studies consistently demonstrate that a single booster dose administered in previously vaccinated individuals elicits strong anamnestic responses across all vaccine antigens, reflecting effective immunologic priming [90]. Importantly, responses are more robust in adolescents who received both infant and preschool doses compared with those primed only in infancy, suggesting that earlier boosting enhances long-term immune memory [62]. In contrast, vaccine-naïve adolescents require a two-dose primary series to achieve adequate protection [63].

From a programmatic perspective, administration of a booster between approximately 11 and 18 years of age—often concomitantly with other adolescent vaccines—represents a pragmatic strategy to restore protection during a period of increased disease risk. Emerging evidence suggests that a one-dose booster in previously vaccinated individuals induces substantial increases in bactericidal antibody titers and may offer a more feasible approach compared with multi-dose schedules, particularly in terms of adherence and program implementation [90,91]. However, important uncertainties remain regarding the optimal age for boosting, the duration of protection following a booster dose, and whether a single booster is sufficient across all priming schedules. Current recommendations therefore remain heterogeneous across countries and are often guided by local epidemiology and health policy considerations.

Overall, the decision to administer a 4CMenB booster in adolescence should be individualized based on prior vaccination history, epidemiological context, and risk profile, while further studies are needed to better define optimal timing and long-term benefits.

## 6. Limitations of the 4CMenB Vaccine

Although the 4CMenB vaccine has demonstrated substantial effectiveness in preventing IMD, several limitations remain that influence its optimal use and overall public health impact.

A key constraint relates to the antigen-specific mechanism of protection. Unlike polysaccharide-conjugate vaccines, 4CMenB targets a limited number of surface-exposed proteins whose expression varies considerably among circulating *N. meningitidis* serogroup B strains. Consequently, vaccine effectiveness is inherently dependent on both the presence and level of expression of these antigens. Predictive models such as MATS and gMATS consistently indicate incomplete strain coverage, and real-world effectiveness may therefore fluctuate over time and across regions in response to shifts in circulating clonal complexes and antigenic variants [35]. Continuous molecular surveillance is thus essential to monitor potential changes in strain distribution and to detect possible vaccine escape.

Another major limitation concerns the duration of protection. While 4CMenB induces strong initial bactericidal responses, longitudinal studies demonstrate progressive waning of antibody titers, particularly for certain antigens such as fHbp and PorA. In addition, selective pressure on vaccine antigens may influence the long-term population structure of circulating meningococci. The precise duration of clinically meaningful protection remains uncertain, complicating the design of optimal vaccination schedules. This issue is particularly relevant for populations at sustained risk, including individuals with underlying medical conditions. In children primed during infancy, protective antibody levels against some antigens decline substantially within 24–36 months after completion of either 2 + 1 or 3 + 1 schedules. These findings support consideration of booster doses in the preschool period, especially for high-risk groups. Similarly, adolescents who were primed in infancy but did not receive a preschool booster may have suboptimal protection and could benefit from revaccination; however, standardized recommendations addressing this scenario are still lacking.

Importantly, unlike conjugate vaccines against other meningococcal serogroups, 4CMenB appears to have limited and inconsistent effects on nasopharyngeal carriage. As a result, indirect protection through herd immunity is minimal or absent, and the overall population impact of vaccination relies primarily on direct protection of vaccinated individuals. This characteristic has important implications for vaccination strategies, as high coverage in target groups does not necessarily translate into broader community protection.

Programmatic and economic considerations also represent significant challenges. Cost-effectiveness analyses of routine 4CMenB vaccination have yielded heterogeneous results, often depending on local disease incidence, vaccine price, healthcare system structure, and modeling assumptions. Early health technology assessments suggested that routine immunization may not be cost-effective in settings where annual IMD incidence is below approximately two cases per 100,000 population. However, such evaluations may underestimate the broader burden of disease, including long-term sequelae, quality-of-life impairment, and societal costs associated with outbreaks. In addition, the lack of herd immunity and the need for potential booster doses further influence cost-effectiveness estimates. Overall, available evidence indicates a consistent gradient whereby expanding vaccination to additional age groups improves epidemiologic effectiveness but progressively reduces cost-effectiveness from a health-economic perspective. Infant-only vaccination provides the greatest direct clinical benefit and can be cost-effective when broader societal costs are incorporated, with Incremental Cost-Effectiveness Ratios (ICERs) falling substantially in comprehensive models compared with narrow healthcare perspectives; however, under more conservative assumptions, ICERs may remain high [e.g., €316,000/Quality-Adjusted Life-Year (QALY) in France], reflecting the rarity of disease despite severe outcomes [92]. Extending vaccination to infants plus school-age children or adolescents increases the number of cases prevented across age groups, but cost-effectiveness generally worsens because of lower incidence outside infancy and limited herd protection, with adolescent programs alone often showing even higher ICERs (e.g., €717,000–€890,000/QALY). Finally, a comprehensive strategy including infants, schoolchildren, and adolescents yields the greatest overall reduction in cases and deaths at the population level, but this broader coverage substantially increases program costs and typically results in the least favorable ICERs unless assumptions include higher incidence, significant indirect effects, or expanded societal valuation of health benefits [92]. Consequently, economic evaluations remain highly context-specific, and policy decisions must be tailored to national epidemiological and healthcare settings.

Further uncertainties relate to the breadth of protection beyond MenB. Although cross-protection against other meningococcal serogroups and *Neisseria gonorrhoeae* has been suggested, the magnitude and durability of these effects remain incompletely defined and, in some cases, controversial. While observational studies indicated moderate protection against gonorrhea in mostly naive individuals, recent randomized evidence in high-risk groups has shown no effect, highlighting the complexity in interpretation of indirect effects.

Finally, practical considerations such as reactogenicity may influence vaccine acceptance and program implementation. Although adverse events are generally mild and self-limiting, the relatively high frequency of post-vaccination fever—particularly in infants—may affect adherence and requires specific mitigation strategies.

In summary, while 4CMenB represents a major advance in the prevention of MenB disease, its use is constrained by incomplete strain coverage, waning immunity, limited impact on carriage, and context-dependent cost-effectiveness. Addressing these limitations will require ongoing surveillance, optimization of vaccination schedules, and further research to better define long-term protection and broader public health benefits.

## 7. Conclusions

IMD remains a rare but devastating infection characterized by rapid progression, high case-fatality rates, and a substantial burden of long-term sequelae. Despite significant advances in prevention, the epidemiology of *N. meningitidis* continues to evolve, with dynamic changes in serogroup distribution, clonal complexes, and age-specific incidence across regions. In many high-income settings, serogroup B remains the predominant cause of IMD, underscoring the continued need for effective and adaptable preventive strategies.

The introduction of protein-based MenB vaccines, particularly 4CMenB, represents a major milestone in meningococcal disease prevention. Evidence from clinical trials and an expanding body of real-world data consistently demonstrates that 4CMenB induces robust immune responses and provides substantial direct protection against MenB disease across different age groups. Implementation of vaccination programs—especially in infants and in outbreak settings—has led to meaningful reductions in disease incidence, confirming the public health value of this approach.

However, important challenges remain. Vaccine effectiveness is influenced by antigenic variability and differential expression of target proteins, resulting in incomplete and geographically variable strain coverage. In addition, waning immunity over time and the limited impact on nasopharyngeal carriage constrain both the duration of protection and the potential for herd immunity. These factors complicate the design of optimal immunization schedules and highlight the need for carefully considered booster strategies, particularly in populations primed in infancy and entering higher-risk periods such as adolescence. This explains why, although immunization of infants is generally recommended, use of the vaccine in school-age children and adolescents varies significantly among countries. Economic considerations further add complexity, as the cost-effectiveness of routine MenB vaccination is highly dependent on local epidemiology, vaccine pricing, healthcare system characteristics, and assumptions regarding long-term protection and indirect effects. Moreover, although potential cross-protection against other meningococcal serogroups and *Neisseria gonorrhoeae* has been suggested, current evidence remains heterogeneous.

Overall, 4CMenB represents an effective tool for the prevention of MenB IMD, but its optimal use requires a tailored, evidence-based approach that integrates epidemiological surveillance, immunologic data, and programmatic feasibility. Continued molecular surveillance, long-term effectiveness studies, and refinement of predictive coverage models are essential to monitor vaccine impact and guide policy decisions. Future strategies could prioritize: (i) optimized booster schedules and ensure equitable access, (ii) integration of genomic surveillance into policy decisions, and (iii) development of next-generation vaccines with broader and more durable coverage, although further evidence is needed.

## Figures and Tables

**Table 1:** Key epidemiological features of invasive meningococcal disease (IMD) and serogroup B (MenB).

Invasive Meningococcal Disease (IMD)		
Domain	Findings/Patterns	Notes/Examples
Global incidence	Low in high-income countries; high in low-resource settings	EU/EEA: 0.4/100,000 (2023); Sub-Saharan Africa: up to 1000/100,000 during outbreaks
Age distribution	Peak in infants; secondary peaks in adolescents and older adults	Infants: 6.5/100,000; 1–4 years: 1.2; 15–24 years: 0.8
High-risk groups	Increased susceptibility independent of age	Complement deficiencies, asplenia, microbiologists, outbreak exposure, military recruits, students
Geographic variability	Marked regional differences in serogroup distribution	MenA (Africa), MenB (Europe/USA), MenX (sub-Saharan Africa)
Clonal complexes	Few hypervirulent lineages drive most IMD	CC11 (MenC/W), CC32/41/44/269 (MenB), CC23 (MenY)
Temporal trends	Fluctuating incidence	Driven by vaccination, strain emergence, behavior, environment
Vaccination impact	Reduction in targeted serogroups	Near-elimination of MenC and MenA in vaccinated populations
COVID-19 impact	Decline followed by rebound	Due to non-pharmaceutical interventions and subsequent immunity debt
Surveillance limits	Heterogeneity across regions	Different case definitions and reporting systems
**Serogroup B (MenB)**		
**Domain**	**Findings/Patterns**	**Notes/Examples**
Global distribution	Endemic in high-income regions; rare elsewhere	Europe, Americas, Australia; rare in China/India
Pre-pandemic trends	Overall decline with variability	Europe: 0.69 → 0.30/100,000 (2008–2017)
Contribution to IMD	Leading cause in many countries	~64% Europe; ~60% USA
High-incidence countries	Limited number of settings	New Zealand, Ireland (>2/100,000)
Clonal complexes	Few lineages dominate	CC32, CC41/44, CC269; regional variants
Vaccination impact	Rapid reduction where implemented	England: ~50% reduction in first year
COVID-19 effect	Decline then resurgence	England: 0.16 → 0.27/100,000
Post-pandemic trends	MenB drives rebound in many regions	Europe, South America, Australia
Regional variation	Other serogroups dominate in some countries	USA (C, Y); France/Chile (Y, W)
Policy influence	Vaccination strategies shape epidemiology	Broader coverage alters age-specific patterns

**Table 2:** National recommendation categories.

Policy Category	Representative Countries	Typical Schedule
Universal for infants, children, adolescents, plus high-risk groups	Australia; Austria; Belgium; Brazil; Canada; Cyprus; Czech Republic; Hungary; Portugal	Infants: 2 + 1; Adolescents: 2 doses; High-risk: 2 doses + booster at 1 year and annual boosters
Infants and high-risk groups (some include routine childhood doses)	Chile; France; Greece; Ireland; Israel; Italy; Lithuania; Poland; San Marino; Spain; United Kingdom	Infants: 2 + 1; High-risk: 2 doses + booster at 1 year and annual boosters
Adolescent recommendation based on shared clinical decision-making	United States	Adolescents: 2 doses (ages 16–23, preferred 16–18); High-risk: 2 doses + booster at 1 year and annual boosters
High-risk groups and outbreak control only	Argentina; Croatia; Finland; Germany; Luxembourg; New Zealand; Norway; Slovenia; Sweden; Uruguay	Outbreak/high-risk: 2 doses; boosters as indicated

**Table 3:** Immunogenicity and safety of 4CMenB vaccine.

Domain	Key Findings	Details/Notes
Infant immunogenicity (3 + 1)	Strong bactericidal responses	95–100% achieve protective titers post-booster; high responses to fHbp, NadA, PorA
Reduced schedules (2 + 1)	High initial seroprotection	98–100% for fHbp/NadA/PorA; 48–77% NHBA; memory response after booster
Antibody persistence	Antigen-specific waning	Durable for NadA; decline for fHbp and PorA over 2–3 years
Booster effect	Strong anamnestic response	97–100% fHbp/NadA; 83–100% PorA; 75–100% NHBA
Toddler vaccination	Limited persistence without booster	Booster restores protection in most children
Children/adolescents	Robust initial response	Persistence varies; better for NadA/NHBA than fHbp/PorA
Long-term immunity	Partial persistence up to years	Protection detectable up to 4–7.5 years for some antigens
Adolescent booster	Dependent on prior schedule	Stronger response if primed in infancy + preschool
Reactogenicity	Common but mild/moderate	Local reactions 25–45%; systemic symptoms frequent
Fever	Most relevant adverse event	Up to 60% infants; higher with co-administration
Mitigation strategies	Effective without reducing immunogenicity	Paracetamol reduces fever incidence

**Table 4:** Real-world effectiveness and impact of 4CMenB vaccine.

Setting/Country	Population	Study Design	Key Findings (VE/VI)	Notes
England	Infants	National observational	~75% reduction; VE ~53% (primary) and ~59% (booster)	High coverage (>90%); sustained > 5 years
Italy	Infants	Regional observational	VE > 90%; VI varies by schedule	Earlier start (2 months) more effective
Spain	Children < 5 years	Case–control	VE 71% MenB; 76% overall IMD	No cases in vaccinated with covered strains
South Australia	Adolescents	Before–after Vaccine effectiveness	Reduction 71–83.5%	Adolescent program impact
Canada (outbreak)	≤20 years	Mass campaign	Incidence 11.4 → 0.4/100k; VE ~79%	High coverage (83%)
USA (college outbreaks)	Young adults	Outbreak response	No further cases post-vaccination	2-dose campaigns effective
Carriage studies	Adolescents/young adults	Observational/RCT	No significant reduction in carriage	Limited herd immunity
Cross-protection	Infants	Surveillance	~69% reduction MenW	Potential broader antigen effect
*Gonorrhea* studies	Adults	Case–control/RCT	~30% short-term VE; no effect in RCT	Uncertain/controversial effect

IMD, invasive meningococcal disease; RCT, randomized controlled trial; VE, vaccine effectiveness.

## Data Availability

No new data were created or analyzed in this study.
